# Supplementary material for: Calibrating agent-based models to tumor images using representation learning
Source: PLoS Comput Biol. 2023 Apr 21;19(4):e1011070. doi: 10.1371/journal.pcbi.1011070 (PMC10156003; doi:10.1371/journal.pcbi.1011070)
Supplement: Attachment — Submitted filename: Review.docx [file pcbi.1011070.s001.docx]

# Summary

The authors present an innovative technique for fitting agent-based models (ABMs) from images by using machine learning. This is an urgent need in the modeling community as ABMs remain very difficult to parameterize. The authors suggest creating a map from ABM parameter space to a low-dimensional representation in the following way: run the ABM with those parameters and map the image at a specified timepoint into a neural network. This neural network is trained on ABM images, minimizing the distance between rotations and reflections of a single image. Then, to constrain ABM parameters from imaging data, the image is processed and subsequently fed through the network into the representation space. There, a distance metric is used to find ABM parameters that map close to the projected image.

# Major comments

Lines 81-83: “in order to **properly** estimate unknown parameters, ABMs of tumor growth **must** be compared to tumor images…” [emphasis added]. This came across more maximalist than I felt necessary. I would like to ask the authors to consider if they want to present their method to the community as useful or if they want to go further and say it’s necessary.

I have some questions regarding the *Data processing* section:

- ABM and tumor images are often not as grid-like as Figure 2A suggests. Are there additional details the authors can provide for this step? I would benefit from comments in the Github code and a zoomed in example from Figure 2B or the workflow with the tumor image from Figure 5B.
- Would it be possible to overlay a grid on the original image and compute the covered area in each channel?
- The authors are clear that the first step only applies to tumor images. Is it true that from the second step (Line 148) on that these are applied to both ABM and tumor images?

On the genetic algorithm:

- Why is the genetic algorithm part not included as part of the method? Do the authors expect the method to work without the GA?
- How do the parameter estimates change across the fitting steps? For example, how do the ranges for T cell killing probability vary if we stop at Fitting step n for n in 1:30?

Lines 290-291: What complexity is added by the living cells that prompts the authors to disregard them? Is it an issue of image size? Complexity of their spatial pattern? Which cells in the image in Figure 5B (top) are alive and which are dead?

Lines 304-306: This seems like a major drawback of the method as it seems to require extensive user input which could introduce bias. The authors should mention this in the Discussion.

# Minor comments

The README.md on Github does not indicate a file which handles the data processing of tumor images. I think they would be processed by the same functions that process model simulations, but the README.md specifies those functions are for model simulations.

Line 86: I ask the authors to consider moving “often” in this way: “For ABMs of tumors, ~~often~~ simple qualitative comparisons are **often** performed…”

Line 137: This “different” method is one the reader would create for their specific application, correct? Perhaps reword to indicate that the user would be responsible for making this new method.

Line 163-164: At the beginning of this section, you say that the method is specific to your use-case. Why is that reiterated here specifically for this final step?

Lines 163-166: Three consecutive sentences with a “…, which…” statement. I have no problem with this, but some authors/readers do.

Line 204: “Now that we have a neural network…” --> “Now that we have an ensemble of neural networks…”
