# Supplementary material for: Calibrating agent-based models to tumor images using representation learning
Source: PLoS Comput Biol. 2023 Apr 21;19(4):e1011070. doi: 10.1371/journal.pcbi.1011070 (PMC10156003; doi:10.1371/journal.pcbi.1011070)
Supplement: Attachment — Submitted filename: responseToReviewers.docx [file pcbi.1011070.s002.docx]

**Response to Reviewers’ Comments**

We thank the reviewers for their constructive review. Below, we provide a response to each comment (in blue font) and indicate how we have edited the manuscript text to address the comments. Additionally, we have provided a file that highlights the changes made to the manuscript text (“Manuscript text_revised_Track-Changes_Highlighted.docx”). The line numbers below refer to that file.

**Reviewer 1**
The central question I was left with after reading the manuscript is: ‘What ‘low’ dimension is used here? There may be some discussion of this in your earlier contribution on the learning network architecture, but it needs to be discussed here as well. Even the order of magnitude is a bit of a mystery: 3? 30? 300? Perhaps some guidelines/strategy for selecting this dimension (and any constraints on that choice) could be discussed in the introduction and then the specific choices made in examples 1 and 2 could be provided and justified.

We thank the reviewer for their comment and agree that this point has not been fully described. In short, we projected tumor and model images to two dimensions. We acknowledge that it is a challenge to determine the optimal number of dimensions, as more dimensions means a more descriptive projection, but the Euclidean distance becomes less meaningful. Unfortunately, “high” dimensional is subjective, so we simply recommend using a low number of dimensions that still yields meaningful results. From our results, we find that two dimensions was sufficient and allows a straightforward implementation of finding the Euclidean distance between points. We edited the Methods section “Training of the neural network” to state this explanation (Lines 214-216).

On a related note, it would be useful to compare your representation learning results to more standard dimensionality-reduction methods (e.g. PCA), for instance, in terms of the dimension of the reduced description. One would not expect PCA to capture the nonlinear relationships between parameter values and ABM simulation output, but this comparison might help your readers better appreciate your contribution.

We have included text and a new reference in the introduction that shows that neural networks perform better than PCA for image dimensionality reduction (Line 100-102). In this way, we keep the focus on the application of our approach to ABM calibration.

Training: It would be good to expand on how to approach training in a more ‘realistic’ scenario where there is an ensemble of experimental images available. It seems that this that case it would be advisable to train on images as well as simulations. Can you speak to any challenges or opportunities that might present?

We thank the reviewer for this point. We agree that it may be possible to generate multiple experimental images; however, this will still likely be a small dataset that is not extensive enough to use for fitting. It is unlikely that an experiment will yield on the order of 1000 images that enable good training of the neural network. Thus, we propose to train only on model simulations for consistency with the method. We have expanded the Methods section “Training of the neural network” to better justify the sole use of simulations (Lines 207-212).

More specifically, for the training described in the manuscript: Are the initial conditions of the simulations widely sampled like the parameter values, or are they fixed to be consistent with the conditions of the experiments? How may training simulations were generated?

That is an excellent point, which we have expanded upon in the Methods section “Generation of training data” (Lines 195-197). In this paper, we make the assumption that initial conditions are known, easing the development of the comparison method, but note that in cases where the initial conditions are unknown, it could be beneficial to sample (and then fit) the initial conditions.

Minor points:

Intro: may want to also highlight the fact that (most) ABM formulations are stochastic, which further complicates calibration.

We agree that the stochastic nature of ABMs can impede model calibration and have mentioned this point in the introduction (Line 73).

Line 25: it’s implied that ‘time-course data’ is non-spatial, which is certainly most commonly the case, but time-lapse microscopy data isn’t captured here. Perhaps add the modifier ‘aggregate’ to clarify.

We have clarified this point (Line 25).

Line 93: “To this end, we endeavored to develop a method for quantitatively comparing experimental tumor images to ABM simulations as a single value.” The phrase ‘as a single value’ is hard to interpret here. Maybe this could be reworded something like ‘To this end, we endeavored to develop a method for establishing a scalar measure for comparison of experimental tumor images and ABM simulations.”

We thank the reviewer for this suggestion and have implemented it in the revised manuscript (Line 94).

Line 166 “The second is that it converts discrete cell locations into regions of cell density, allowing us to compare across spatial scales.” You’ve used the verbs ‘convert’ and ‘reduce’ in this description, but it may be useful to use the term ‘aggregate’ to better convey the meaning. Also, there’s some ambiguity in using ‘grid’ to refer to the lattice and to lattice-points. E.g. in Fig 2 caption ‘grids are then reduced to a smaller size’ maybe could be reworded something like: ‘grid-points are then aggregated, resulting in a coarser grid, where values correspond to local densities.’

We have implemented this change (Line 172).

Finally, Fig 2B: The figure doesn’t suggest that the size has changed (as in A), but rather a coarser grid has been applied (so the image is smaller in terms of pixel dimensions, but not inches on the page).

We added a note in the figure caption that, while B (center) and B (right) are the same size visually, B (right) has fewer pixels/grid spaces (Lines 226-227). We thank the reviewer for helping to clarify this point.

Line 179: “Following image processing, the next step of our method is to generate the model simulations needed to train the neural network.” This wording may be misleading as could be understood to suggest that images are processed before they are generated. Perhaps you mean to say ““Following the establishment of an image processing protocol, the next step of our method is to generate the model simulations needed to train the neural network (to which that image processing protocol will be applied).

We have edited the text to clarify this point (Line 189-191).

Line 209: perhaps reword: “We then take the n closest simulations to the image and examine their parameter *values*

We have made this change (Line 236).

Caption fig 3: The wording here suggests that the choice of ‘nearby’ parameter value sets is based on a distance threshold, whereas in the text it’s indicated that the nearest ‘n’ parameter value sets are selected. Either are reasonable, but the wording can be made consistent.

We have reworded the legend to include “n closest simulations” (Line 242)

Line 219: ABM description. You’ve made clear that this simulation is being run just for illustration, but it’s still a bit concerning not to have a full description of the ABM details. Perhaps the code could be provided? That would ensure that a reader would be in a position to replicate your results.

We agree that it is important for readers to implement our method and reproduce the results. The code for both models has been added to the GitHub repository for this paper. We also mention this in the manuscript text (Lines 447-448).

Line 277: best to describe this as the endpoint of the simulation, yes?

We agree and have made this change (Line 309).

Line 280: “the based parameters” -> ‘base parameters’, ? You may consider using the more specific term ‘nominal’ instead of ‘base’ for your reference simulation. Likewise in table 1 the ‘base parameter value’ is inconsistently referred to as ‘actual’

We have edited the manuscript to use “nominal parameters”, rather than “base parameters” (Lines 311 and 312) and have replaced “actual” with “nominal” in Table 1.

Table 1: the line between maximum and mean is oddly placed. Not clear why it’s there.

We have moved the line reporting the mean to be after the reported minimum values, both in Table 1 and Table 2.

Also, it might be more useful to report the CoV instead of s.d., especially here where the number format is not consistent.

We now report the coefficient of variance rather than standard deviation.

Finally, it would be interesting to see the max/min search range that you’re exploring (to get an idea of how narrow the search space is, and whether any of the best fits are worryingly close to those bounds). This is especially important in example 2 where the manual choice of parameter search region muddies the description of the search.

We agree that these are interesting questions to address in the context of model building and calibration. However, we chose not to include this in the manuscript in order to maintain focus of this study on implementation of representation learning as an objective function for tumor ABMs. Future work will provide an in-depth description of critical aspects of model development, including interactions included, parameter sensitivity analysis, and results of parameter estimation. We have included a statement about this in the Discussion (Lines 421-427).

Line 295: “of approximately 3,000 microns in diameter or 150 cells” Ambiguous wording, I presume this means “of approximately 3,000 microns (i.e. 150 cells) in diameter”

We have edited the text to clarify this point (Line 333).

Example 2: the ‘across scales’ fitting argument is a little hard to follow. As the ABM designer, you can select how coarse to set your grid, so you could interpret your model as clusters of cells at each grid-point. Is that the interpretation being used here?

We are aiming to demonstrate that our approach works well even when the size of the tumors simulated by ABMs are different from the size of the tumor images used for calibration. Other studies have noted that some parameters need to be scaled due to the fact that modeled ABMs are usually smaller than actual tumors. Here, we note that, by coarse-graining cell locations as we describe in the processing step, we can directly compare images/simulations of different spatial scales. We have edited the manuscript to better state this point (Lines 340-342).

Line 302: manual setting of bounds is fine, but is inconsistent with your previous description of the algorithm (bounds set in terms of distance in the low-dimensional representation). Were these both used? Or the latter not employed?

We thank the reviewer for raising this point. We followed the same procedure with narrowing the bounds based on the low-dimensional representation. In addition, we also manually set some parameter bounds to more quickly arrive at the final fitted parameters. We have edited the manuscript to state this point (Lines 344-354). We also mention this in the limitations paragraph, when discussing the computational expense of our approach (Lines 417-419).

Line 331: “We compare the images and model simulations using representation learning to project model simulations and tumor images into low-dimensional space and then calculating the distance between the two.” Grammar: ‘and then calculate…’

We have fixed this grammatical error.

**Reviewer 2**

I would suggest changing the title from “fitting” to “validating” or “calibrating and validating.” In my experience, non-ABM people often use the term fitting pejoratively. I leave it up to you, this has just been my experience in the field.

We thank the reviewer for this suggestion to better capture the focus of this work. We have replaced “fitting” with “calibrating” in the title.

It would be useful to cite other examples of GA’s being used for parameter exploration in ABMs.

We used a GA due to ease of implementation on our computing cluster, but other estimation algorithms can certainly be used instead. We have added a statement to mention this point (Line 297-300).

On line 260, you state, “The model is stochastic, so we cannot expect to fit the parameters perfectly.” Is there a range of parameters that generate equivalent results over some number of stochastic replicates? Or is the supposition that there is a unique best fit?

We report the fitted parameter values at the end of the genetic algorithm and do not assume there is a single best fit. To avoid confusing readers, we have edited the manuscript to more clearly state the goal of obtaining fitted parameters close to the set values (Lines 287-289).

On line 299, the authors state “and it is currently impossible to simulate tumors on that scale.” This is debatable are there are biomedical ABMs out there that operate at anatomic scale and incorporate tens of billions of cells.

We appreciate this comment and have changed the wording (Line 336-337).

If there are any plans for extending this to 3d tumors, that should be included in the discussion.

Ideally, our agent-based modeling framework would be extended to 3D; however, this is of lower priority at this moment due to the computational expense. Additionally, it would be difficult to obtain 3D-resoved tumor images to which the model could be calibrated. Thus, we have not mentioned this future work in the manuscript.

As a matter of style, superscript citations typically come after periods. There are a few minor typos also (line 370: “computational expensive.”)

We have addressed small grammatical errors throughout and will update the citations based on the journal style.

**Reviewer 3**

Lines 81-83: “in order to **properly** estimate unknown parameters, ABMs of tumor growth **must** be compared to tumor images…” [emphasis added]. This came across more maximalist than I felt necessary. I would like to ask the authors to consider if they want to present their method to the community as useful or if they want to go further and say it’s necessary.

We have edited the wording to better emphasize the utility of our approach (Line 82-83).

I have some questions regarding the *Data processing* section:

- ABM and tumor images are often not as grid-like as Figure 2A suggests. Are there additional details the authors can provide for this step? I would benefit from comments in the Github code and a zoomed in example from Figure 2B or the workflow with the tumor image from Figure 5B.

Figure 2A is meant for visualizing the process, which Figure 2B shows the application on a more realistic model output. We agree with the reviewer that model and experimental images do not strictly follow a grid-like layout. We acknowledge that we do lose some of the spatial detail when cells are discretized to a grid from their continuous coordinates and then aggregated into cell densities when we shrink the grids. However, this is necessary in order to process both simulations and tumor images into a comparable format. We have edited the manuscript to better state this point (Lines 183-186).

- Would it be possible to overlay a grid on the original image and compute the covered area in each channel?

The reviewer is correct – this would be an alternate way to process the image. We have edited the manuscript to state that our processing method is not the sole method that may be used (Line 140-142).

- The authors are clear that the first step only applies to tumor images. Is it true that from the second step (Line 148) on that these are applied to both ABM and tumor images?

This step is also applied to tumor images and ABM simulations. We have edited the manuscript to better state this point (Line 153-154).

On the genetic algorithm:

- Why is the genetic algorithm part not included as part of the method? Do the authors expect the method to work without the GA?

We did not specifically mention the GA in the Methods, since the choice of estimation algorithm is not part of the overall methodology we present. We used a GA due to ease of implementation on our computing cluster, but other estimation algorithms can certainly be used instead. We have added a statement to mention this point (Line 297-300).

- How do the parameter estimates change across the fitting steps? For example, how do the ranges for T cell killing probability vary if we stop at Fitting step n for n in 1:30?

We agree that these are interesting questions to address in the context of model building and calibration. However, we chose not to include this in the manuscript in order to maintain focus of this study on implementation of representation learning as an objective function for tumor ABMs. Future work will provide an in-depth description of critical aspects of model development, including interactions included, parameter sensitivity analysis, and results of parameter estimation. We have included a statement about this in the Discussion (Lines 421-427).

Lines 290-291: What complexity is added by the living cells that prompts the authors to disregard them? Is it an issue of image size? Complexity of their spatial pattern? Which cells in the image in Figure 5B (top) are alive and which are dead?

In analyzing the in vitro tumor image, we found that there was some overlap between the live and dead cells; however, this phenomenon was not captured by the model. Rather than increase the complexity of the model and add additional parameters, we elected to remove the living cells. This did not hamper the ability of the method to accurately capture the density of cells. We have clarified this point in the text (Lines 326-329).

Lines 304-306: This seems like a major drawback of the method as it seems to require extensive user input which could introduce bias. The authors should mention this in the Discussion.

We have revised the manuscript to better state manually refining the parameter bounds (Lines 344-354), and we have added this as limitation in the Discussion (Lines 417-419).

The README.md on Github does not indicate a file which handles the data processing of tumor images. I think they would be processed by the same functions that process model simulations, but the README.md specifies those functions are for model simulations.

We sincerely appreciate the reviewer looking at the GitHub files to help promote implementation and reproducibility of our work. We have updated the README file to specify the files that perform image processing.

Line 86: I ask the authors to consider moving “often” in this way: “For ABMs of tumors, ~~often~~ simple qualitative comparisons are **often** performed…”

We have implemented this change (Line 86).

Line 137: This “different” method is one the reader would create for their specific application, correct? Perhaps reword to indicate that the user would be responsible for making this new method.

We have included additional text to clarify this point (Lines 140-142).

Line 163-164: At the beginning of this section, you say that the method is specific to your use-case. Why is that reiterated here specifically for this final step?

We have removed this statement to better reflect the applicability of our approach (Line 168-169).

Lines 163-166: Three consecutive sentences with a “…, which…” statement. I have no problem with this, but some authors/readers do.

We have edited the text to remove this repetitive sentence structure (Lines 169-172).

Line 204: “Now that we have a neural network…” --> “Now that we have an ensemble of neural networks…”

We have implemented this change (Line 230).
